# Supplementary material for: Double superionicity in icy compounds at planetary interior conditions
Source: Nat Commun. 2023 Nov 21;14:7580. doi: 10.1038/s41467-023-42958-0 (PMC10663582; doi:10.1038/s41467-023-42958-0)
Supplement: Supplementary file 1 — Supplementary Information [file 41467_2023_42958_MOESM1_ESM.pdf]

Supplementary Information

Double Superionicity in Icy Compounds at Planetary Interior Conditions

Kyla de Villa[1], Felipe González-Cataldo[1] and Burkhard Militzer[1,2]

[1]Department of Earth and Planetary Science, University of California, Berkeley, California, 94720, USA.
[2]Department of Astronomy, University of California, Berkeley, California, 94720, USA.

Contents

1. Benchmarks of DFT **3**
1A. K-point Convergence 3
1B. Cell Size Convergence 5
1C. PBE Pseudopotentials 5

2. Simulations using DFT **6**
2A. Bader Charge Analysis 6
2B. Visualization of Double Superionicity in H_3NO_4-$C2_1$, HCNO-$Pca2_1$-II, and CH_2N_2-$Pna2_1$ 7
2C. Example NPT Simulation 9
2D. Two-phase Simulation Using NVT 10

3. Machine Learning **12**
3A. Training, Exploration, Labelling 12
3B. Benchmarks of Machine Learning Potentials 14

1. Benchmarks of DFT

1A. K-point Convergence

T(K)	N_{atoms}	k-point mesh	Energy (eV)	Pressure (eV/A)	Stress Tensors (X Y Z)
500	96	1x1x1	-211.918	5064.91	5097.52 5092.07 5005.15
500	96	2x2x2	-214.527	5036.13	5050.58 5084.58 4973.24
500	96	3x3x3	-214.533	5036.05	5050.67 5084.54 4972.92
500	96	4x4x4	-214.533	5036.04	5050.66 5084.54 4972.92
2000	96	1x1x1	-194.85	5199.34	5264.25 5184.45 5149.32
2000	96	2x2x2	-197.242	5173.41	5220.65 5182.83 5116.73
2000	96	3x3x3	-197.249	5173.31	5220.78 5182.76 5116.39
2000	96	4x4x4	-197.25	5173.3	5220.77 5182.76 5116.38
4000	96	1x1x1	-152.218	5431.54	5534.75 5399.58 5360.28
4000	96	2x2x2	-153.588	5413.01	5467.98 5422.71 5348.36
4000	96	3x3x3	-153.6	5412.82	5467.75 5422.58 5348.12
4000	96	4x4x4	-154.03	5413.3	5454.97 5419.17 5365.75
6000	96	1x1x1	-83.5813	5793.89	5905.21 5715.47 5761
6000	96	2x2x2	-85.6142	5771.81	5821.87 5754.76 5738.8
6000	96	3x3x3	-85.5908	5772.2	5822.84 5755.88 5737.86
6000	96	4x4x4	-85.5901	5772.2	5822.86 5755.74 5738.02

Table S1 K-points convergence tests for H_3NO_4-$P2_12_12_1$ 96 atom cell at 6.033 g/cm^3. Each data point is the average of 5 configurations.

T(K)	N_{atoms}	k-point mesh	Energy (eV)	Pressure (eV/A)	Stress Tensors (X Y Z)
500	144	1x1x1	-398.676	4010.29	4035.03 3954.43 4041.4
500	144	2x2x2	-398.908	4005.62	4053.86 3934.24 4028.78
500	144	3x3x3	-398.909	4005.62	4053.88 3934.22 4028.77
500	144	4x4x4	-398.909	4005.63	4053.89 3934.22 4028.78
2000	144	1x1x1	-372.467	4149.15	4178.25 4089.2 4180
2000	144	2x2x2	-372.528	4145.98	4201.26 4067.45 4169.22
2000	144	3x3x3	-372.53	4145.97	4201.26 4067.43 4169.2
2000	144	4x4x4	-372.53	4145.97	4201.27 4067.44 4169.2
4000	144	1x1x1	-313.848	4381.16	4381.88 4333.89 4427.7
4000	144	2x2x2	-313.469	4380.52	4419.1 4307.91 4414.53
4000	144	3x3x3	-313.477	4380.45	4419 4307.79 4414.54
4000	144	4x4x4	-313.477	4380.45	4419.02 4307.79 4414.55
6000	144	1x1x1	-211.2	4754.02	4678.93 4825.6 4757.54
6000	144	2x2x2	-211.316	4755.59	4730.18 4796.5 4740.08
6000	144	3x3x3	-211.303	4755.67	4730.39 4796.61 4740.03
6000	144	4x4x4	-211.302	4755.69	4730.38 4796.61 4740.07

Table S2 K-points convergence tests for H_3NO_4-$C2_1$ 144 atom cell at 5.644 g/cm^3. Each data point is the average of 5 configurations.

T(K)	N_{atoms}	k-point mesh	Energy (eV)	Pressure (eV/A)	Stress Tensors (X Y Z)
500	128	1x1x1	-526.446	5071.44	5120.8 5047.24 5046.27
500	128	2x2x2	-527.443	5062.66	5079.43 5049.81 5058.75
500	128	3x3x3	-527.442	5062.66	5079.7 5049.59 5058.7
500	128	4x4x4	-527.442	5062.66	5079.69 5049.6 5058.7
2000	128	1x1x1	-497.245	5148.1	5168.76 5087.4 5188.15
2000	128	2x2x2	-498.072	5141.33	5126.61 5090.65 5206.72
2000	128	3x3x3	-498.075	5141.26	5126.87 5090.36 5206.54
2000	128	4x4x4	-498.075	5141.26	5126.86 5090.38 5206.54
4000	128	1x1x1	-452.965	5372.51	5498.1 5287.72 5331.72
4000	128	2x2x2	-453.878	5366	5457.01 5288.13 5352.85
4000	128	3x3x3	-453.888	5365.79	5456.95 5287.71 5352.7
4000	128	4x4x4	-453.887	5365.8	5456.95 5287.76 5352.68
6000	128	1x1x1	-402.841	5526.42	5593.2 5506.87 5479.18
6000	128	2x2x2	-402.955	5526.15	5550.29 5514.52 5513.65
6000	128	3x3x3	-402.979	5525.87	5550.83 5513.31 5513.46
6000	128	4x4x4	-402.979	5525.87	5550.82 5513.3 5513.47

Table S3 K-points convergence tests for HCNO-*Pca2*$_1$ 128 atom cell at 5.977 g/cm^3. Each data point is the average of 5 configurations.

T(K)	N_{atoms}	k-point mesh	Energy (eV)	Pressure (eV/A)	Stress Tensors (X Y Z)
500	160	1x1x1	-647.576	5083.75	5054.86 5100.79 5095.61
500	160	2x2x2	-649.18	5066.45	5079.95 5050.56 5068.86
500	160	3x3x3	-649.184	5066.54	5079.59 5050.83 5069.2
500	160	4x4x4	-649.184	5066.54	5079.59 5050.83 5069.2
2000	160	1x1x1	-612.579	5165.4	5097.84 5190.38 5207.98
2000	160	2x2x2	-614.252	5149.44	5128.57 5136.03 5183.72
2000	160	3x3x3	-614.26	5149.48	5128.17 5136.28 5184
2000	160	4x4x4	-614.26	5149.49	5128.17 5136.28 5184
4000	160	1x1x1	-552.795	5233.83	5137.92 5165.28 5398.28
4000	160	2x2x2	-556.8	5233.11	5225.62 5088.28 5385.4
4000	160	3x3x3	-554.588	5224.14	5188.33 5105.21 5378.88
4000	160	4x4x4	-556.849	5232.48	5223.95 5088.63 5384.87
6000	160	1x1x1	-424.301	5322.2	5219.67 5363.96 5382.96
6000	160	2x2x2	-421.861	5334.44	5288.01 5357.85 5357.45
6000	160	3x3x3	-421.858	5334.49	5288.8 5357.12 5357.55
6000	160	4x4x4	-421.858	5334.49	5288.8 5357.12 5357.56

Table S4 K-points convergence tests for CH_2N_2-*Pna2*$_1$ 160 atom cell at 5.311 g/cm^3. Each data point is the average of 5 configurations.

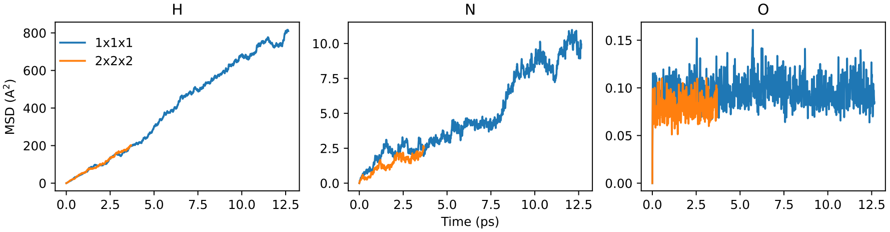

Figure S1 Comparison of MSDs for H_3NO_4-$P2_12_12_1$ at 4500 K when using a 1x1x1 and a 2x2x2 k-point grid.

1B. Cell Size Convergence

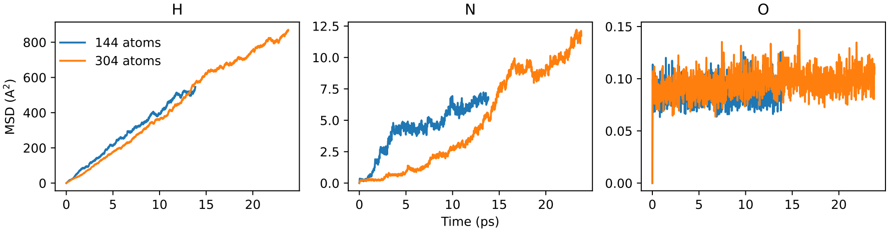

Figure S2 Comparison of MSDs for H_3NO_4-$C2_1$ at 4000 K for 144 and 304 atom cells.

1C. PBE Pseudopotentials

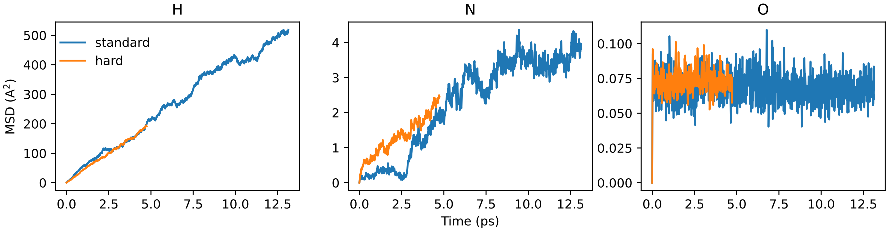

Figure S3 Comparison of MSDs for H_3NO_4-$P2_12_12_1$ at 4000 K when using standard cutoff PBE pseudopotentials and hard cutoff PBE pseudopotentials.

2. Simulations using DFT

2A. Bader Charge Analysis

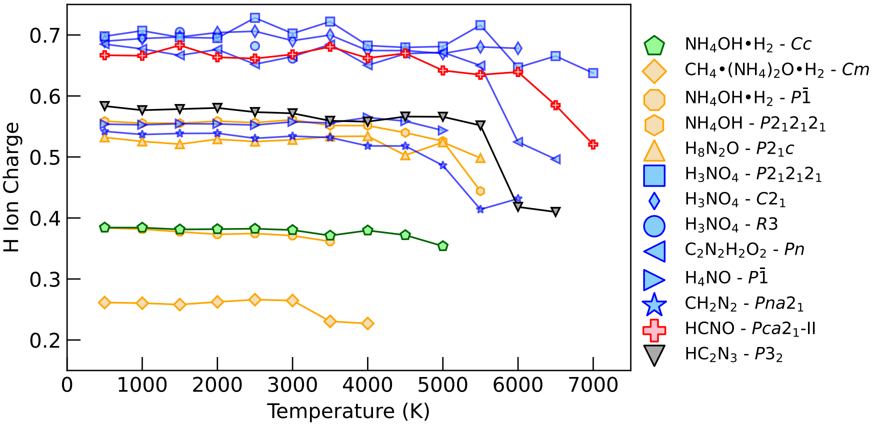

Figure S4 Positive ionic charge of H ions calculated from Bader Charge Analysis for 13 H-C-N-O materials. The average charge of the first 2000 K was used to calculate the diffusivities of the superionic and doubly superionic phases shown in **Fig. 1c** in the main text. In several materials, the electronic environment changes upon melting such that the protons carry more electronic charge.

2B. Visualization of Double Superionicity in H_3NO_4-$C2_1$, HCNO-$Pca2_1$-II, and CH_2N_2-$Pna2_1$

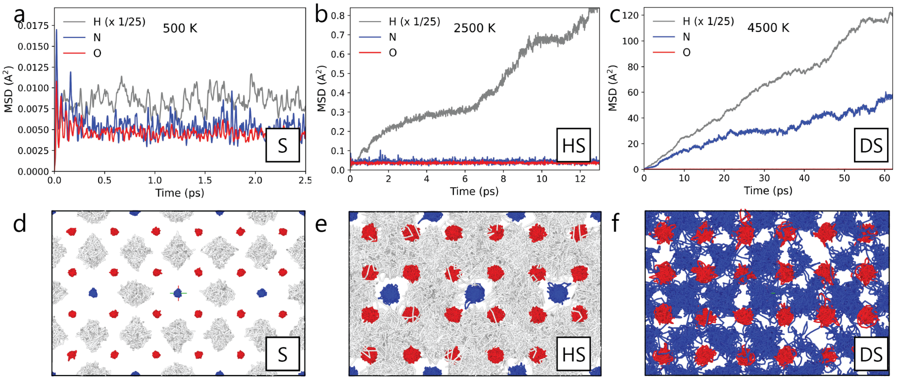

Figure S5 Visualization of H and N Diffusion in H_3NO_4-$C2_1$. a-c Mean squared displacement (MSD) trends for all elements for **a** solid (S), **b** hydrogen superionic (HS), and **c** doubly superionic (DS) phases. For clarity the MSD for H has been multiplied by 1/25 in each plot. **d-f** Atomic trajectories over 5 ps for corresponding temperatures (above). In **f**, H trajectories have been omitted to better visualize N diffusion. All results shown in this figure were derived from DFT-MD simulations.

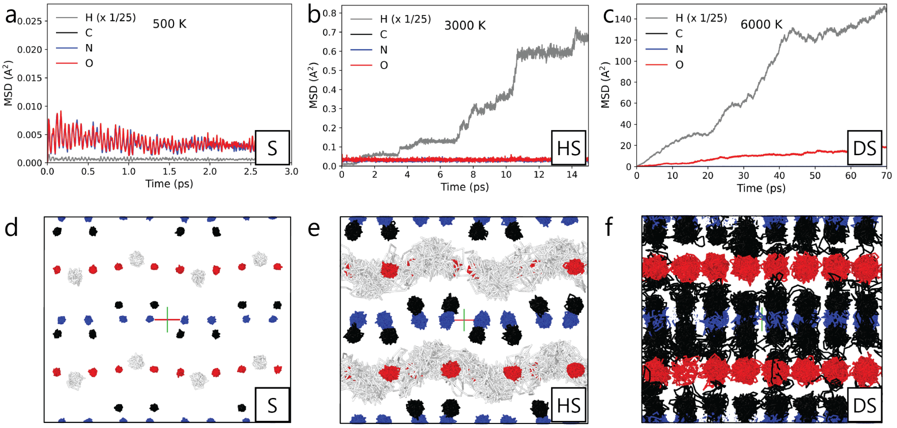

Figure S6 Visualization of H and C Diffusion in HCNO-$Pca2_1$-II. a-c Mean squared displacement (MSD) trends for all elements for **a** solid (S), **b** hydrogen superionic (HS), and **c** doubly superionic (DS) phases. For clarity the MSD for H has been multiplied by 1/25 in each plot. **d-f** Atomic trajectories over 5 ps for corresponding temperatures (above). In **f**, H trajectories have been omitted to better visualize C diffusion. All results shown in this figure were derived from DFT-MD simulations.

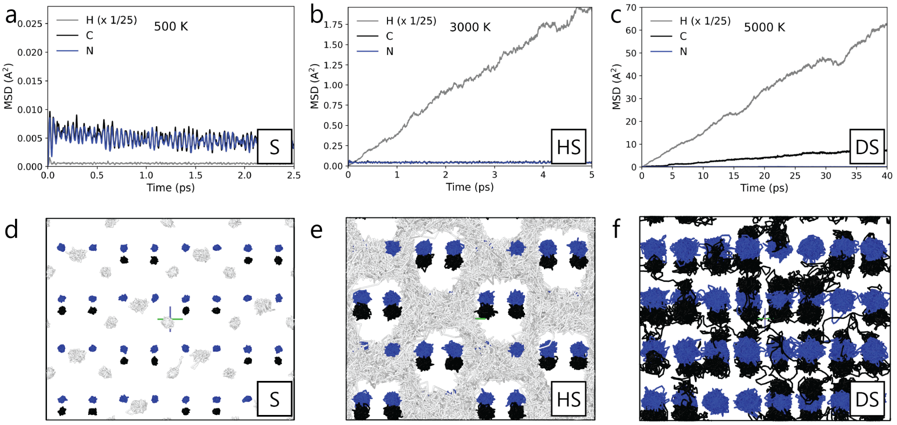

Figure S7 Visualization of H and C Diffusion in CH_2N_2-*Pna2*$_1$. **a-c** Mean squared displacement (MSD) trends for all elements for **a** solid (S), **b** hydrogen superionic (HS), and **c** doubly superionic (DS) phases. For clarity the MSD for H has been multiplied by 1/25 in each plot. **d-f** Atomic trajectories over 5 ps for corresponding temperatures (above). In **f**, H trajectories have been omitted to better visualize C diffusion. All results shown in this figure were derived from DFT-MD simulations.

2C. Example NPT Simulation

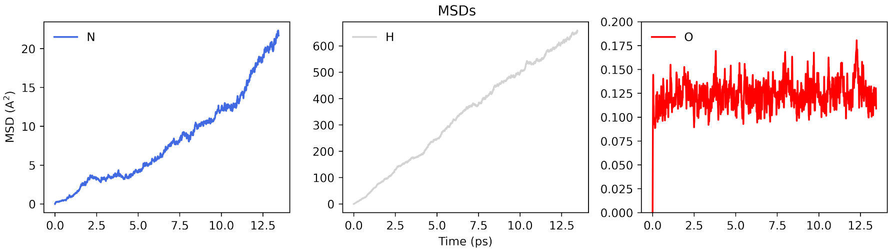

Figure S8 MSDs for NPT simulation of H_3NO_4-$P2_12_12_1$ at 525 GPa and 4500 K. We see that even with a flexible cell, N and H diffuse superionically through a stable lattice of O ions.

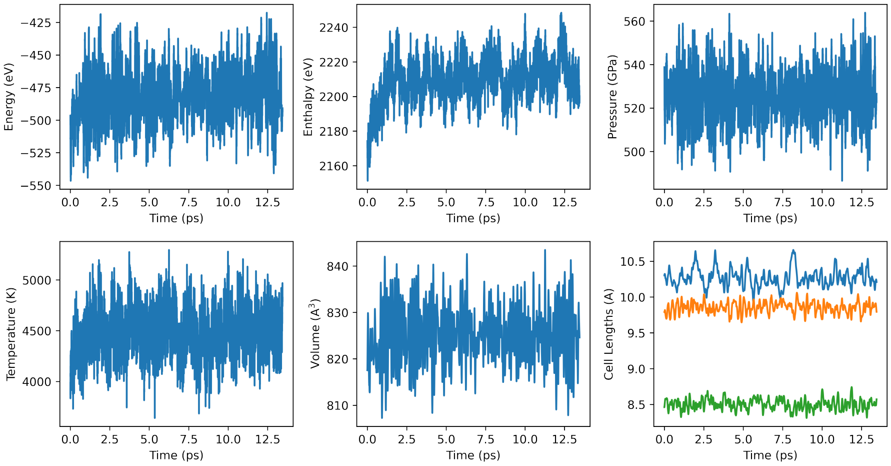

Figure S9 Thermodynamic data for NPT simulation of H_3NO_4-$P2_12_12_1$ at 525 GPa and 4500 K. Energy, volume, and enthalpy all increase as the N ions begin to diffuse, but the cell lengths are stable throughout, indicating that even though the cell is fluctuating, it is stable as H and N both diffuse superionically.

2D. Two-phase Simulation Using NVT

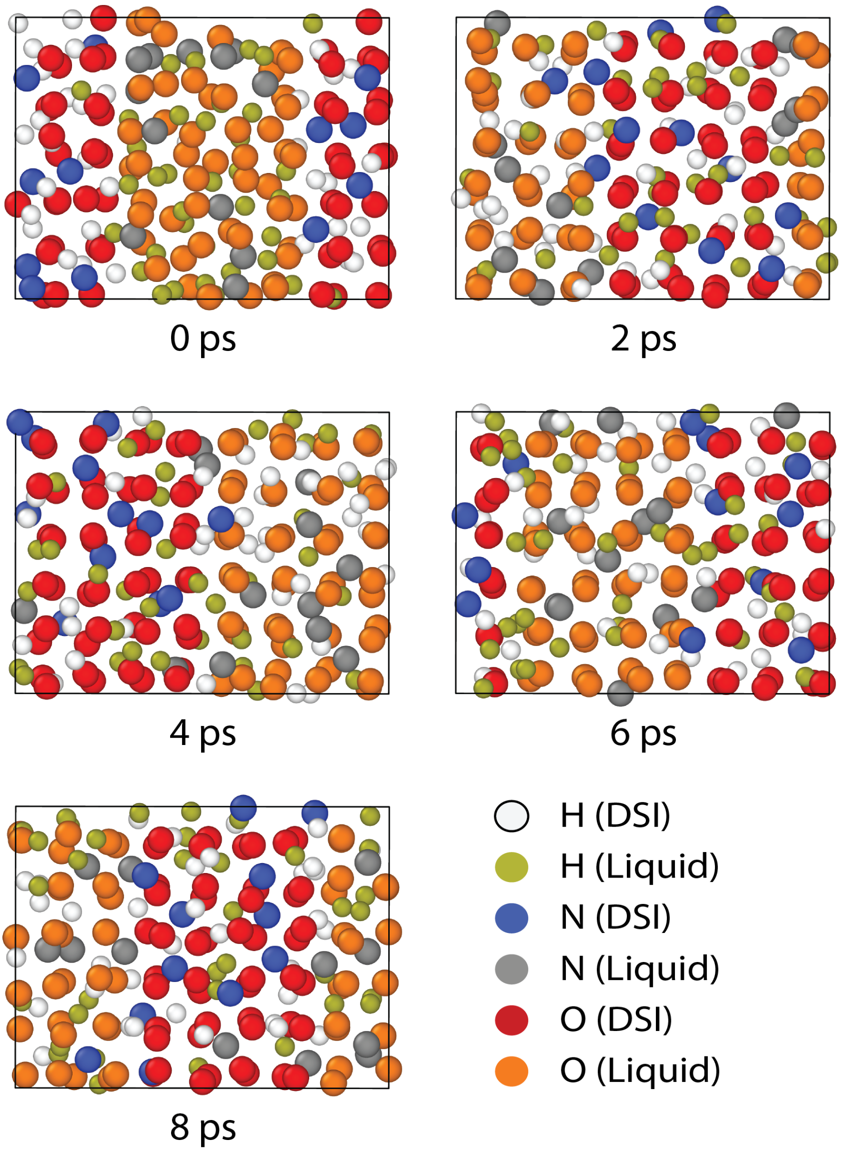

Figure S10 Snapshots of atomic positions of a two-phase simulation performed with DFT-MD at 4000 K and ∼500 GPa for H_3NO_4-$P2_12_12_1$. Atoms are colored based on their original phase. At t=0, a doubly superionic phase (red, white, blue) and a liquid phase (yellow, orange, gray) share two interfaces. Within 2 ps the O ions in the liquid phase crystallize into the same fcc lattice of the DSI phase, while H and N ions originally in each phase continue to diffuse superionically throughout the cell.

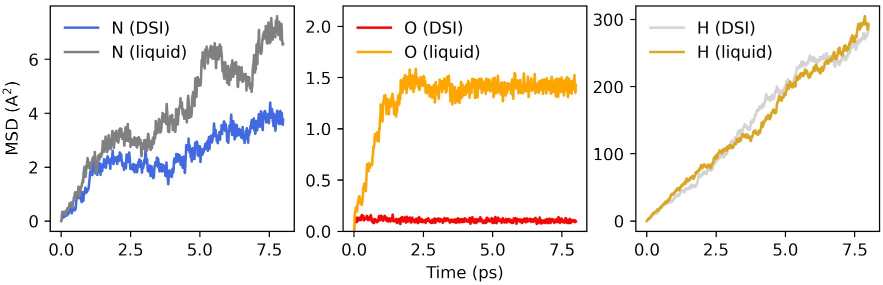

Figure S11 MSD plots of a two-phase simulation performed with DFT-MD at 4000 K and ∼500 GPa for H_3NO_4-$P2_12_12_1$. Trends are colored based on their original phase and match atom colors shown in snapshots in **Figure S10**. The O atoms originally in the DSI phase remain stable throughout the simulation. After ∼2 ps, O atoms originally in the liquid phase begin to freeze, while H and N continue to diffuse superionically throughout the cell.

3. Machine Learning

3A. Training, Exploration, Labelling

Our potentials were trained using an active learning method characterized by a cycle of Initial Data Preparation, Training, Exploration, and Labelling.

Initial Data Preparation: It is necessary to train machine learning potentials on pressures and temperatures higher than one wishes to replicate with ML-MD simulations. If this step is not taken issues may occur, such as very small inter-atomic distances being interpreted by the machine learning potential as having attractive forces between the atoms, resulting in "clumping" of atoms of the same element. To develop our training set, highly accurate DFT-MD-NVT simulations were performed for at least 10,000 steps for 500 K through 1000 K above the melting point, in 500 K increments. For each temperature simulations were performed for a range of pressures (i.e. for H_3NO_4-$P2_12_12_1$ we trained on 450 GPa–550 GPa in 25 GPa increments). 500 equally spaced configurations were pulled from each of these temperature/-density simulations to serve as the initial data set.

Training: Four potentials, differentiated only by random seeds, were initially trained only on DFT-MD data using the Deep Potential for Molecular Dynamics (DeePMD) method [1, 2]. Descriptors for the neural networks were determined using the full relative coordinates (angular and radial) of all atoms within a cutoff radius of 6 Å, with smooth cutoff radii of 5-6 Å. The descriptor was then parametrized using an embedding net of 3 layers (with 25, 50, and 100 nodes). A three layer fitting network, with 240 nodes per layer, was used to map the potential energy surfaces of each system.

Potentials were trained over 1,000,000 steps, with a batch size of 1, an initial learning rate of 10^{-3}, and a final learning rate of 10^{-8}. Our loss function was defined with the following coefficients: energy [initial value=0.02, final value=1], force [1000, 1], and virial [0, 0]. (We found no benefit from training with virial information.)

Exploration: Once trained, one of the four potentials was used to drive NVT molecular dynamics simulations in LAMMPS [3], with a timestep of 0.2 fs. Forces and energies of each configuration were calculated using all four potentials. Model deviations, which indicate the consistency in force and energy predictions between the different potentials, were calculated at each time step. If model deviations were above the training error for a specific configuration, this indicated that the potentials were not well trained at predicting the potential energy surface for this arrangement of atoms, and the accuracy of the machine learning potential would be improved by also training on that configuration.

Following each Exploration stage, we chose new configurations to add to our

data set from those generated with LAMMPS simulations based on the maximum standard deviation of force on any atom in a configuration, given by [4]:

$$\epsilon_t = \max_i \sqrt{\langle \| F_{w,i}(\mathbf{R}_t) - \langle F_{w,i}(\mathbf{R}_t) \rangle \|^2 \rangle} \tag{1}$$

in which w indicates the potential index and i is the atom index within a given configuration $\mathbf{R}_t$. For each atom in a configuration, the forces on the atom were calculated using all four potentials, and the highest standard deviation in forces between these potentials across all atoms in the configuration was determined. In each iteration, 300 random configurations with model deviations slightly above the training error (between 0.1 and 0.3 eV/Å) were added were chosen to be added to the training set in a subsequent training generation.

Labelling: Configurations extracted from LAMMPS trajectories were "labelled" in VASP with the same settings as described for the DFT-MD trajectories, so as to have DFT-accurate energies and forces to train the next generation on. Thereafter, these configurations were added to the existing data set.

Following this cycle of Training, Exploration, and Labelling, a new Training process was initiated with the updated data set. This process was repeated 3 times for each material, until the model deviations were comparable to the average force training errors.

3B. Benchmarks of Machine Learning Potentials

A number of benchmarking analyses can be performed to ensure the consistency of performance between DFT and machine learning potentials (in addition to the comparisons of diffusivities and MSD trends presented in the main text). Below we present a number of such analyses completed for H_3NO_4-$P2_12_12_1$, including root-mean-square-errors (RMSE) from the training of the machine learning potential; force and energy parity plots between DFT and DeePMD; parity plots between the average energy of a simulation predicted by DFT and DeePMD, as well as the variances in energy measured in each simulation; and radial distribution function comparisons between the two simulation methods.

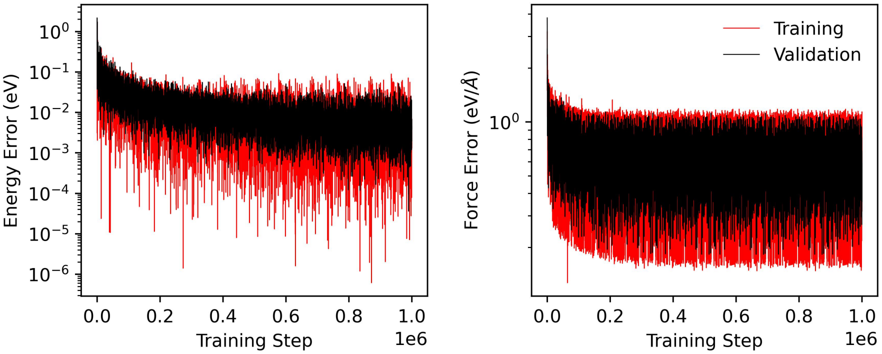

Figure S12 Root-mean-square-error (RMSE) of energy and force for training and validation sets for H_3NO_4-$P2_12_12_1$. This potential was trained on a 288 atom cell and using DFT-MD data from 500 K to 7000 K and 450–550 GPa.

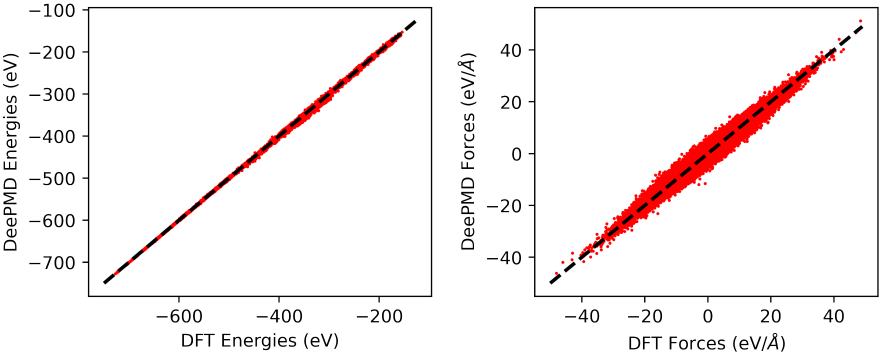

Figure S13 Comparisons of energy and force parities between DFT and DeePMD for H_3NO_4-$P2_12_12_1$. These plots show 15000 energy comparisons and 12960000 force comparisons from 500 K to 7000 K and 450–550 GPa.

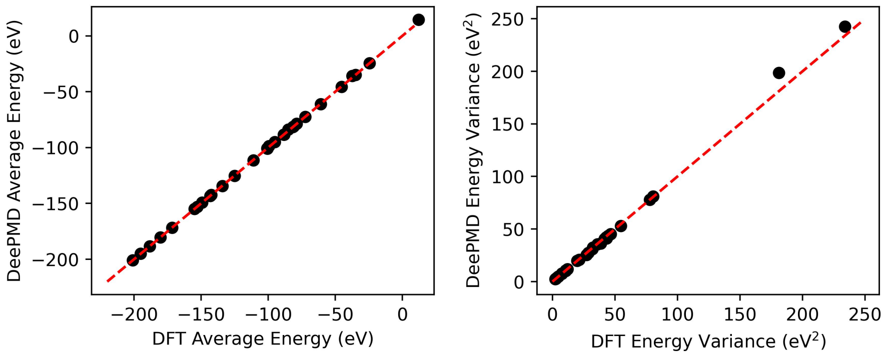

Figure S14 Average energy and variance in energy for DFT and DeePMD for $\mathbf{H_3NO_4}$-$\boldsymbol{P2_12_12_1}$ simulations from 500 K to 7000 K and 450–550 GPa.

After 1,000,000 training steps, the root-mean-squared-errors (RMSE) of the energies of the training and validation data sets were 6.14 and 6.25 meV/atom, and of the forces were 619 and 622 meV/$\AA$. We find these values to be comparable to those listed for similar works on high-pressure ices [5].

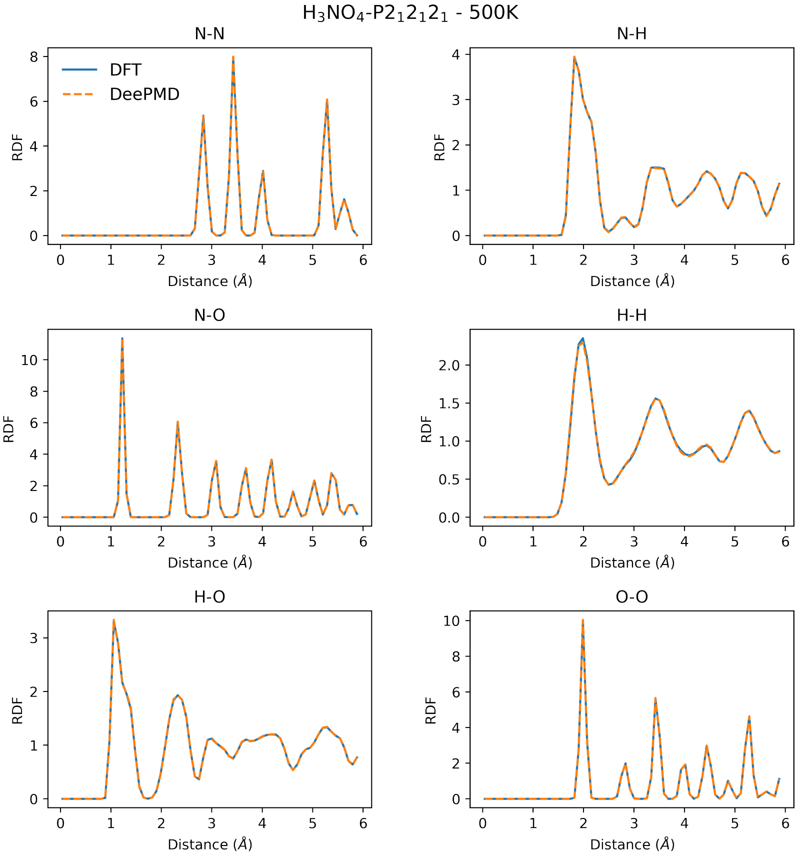

Figure S15 Radial distribution function comparison for DFT and DeePMD for H_3NO_4-$P2_12_12_1$ simulations for the solid phase, 500 K at 6.033 g/cm^3.

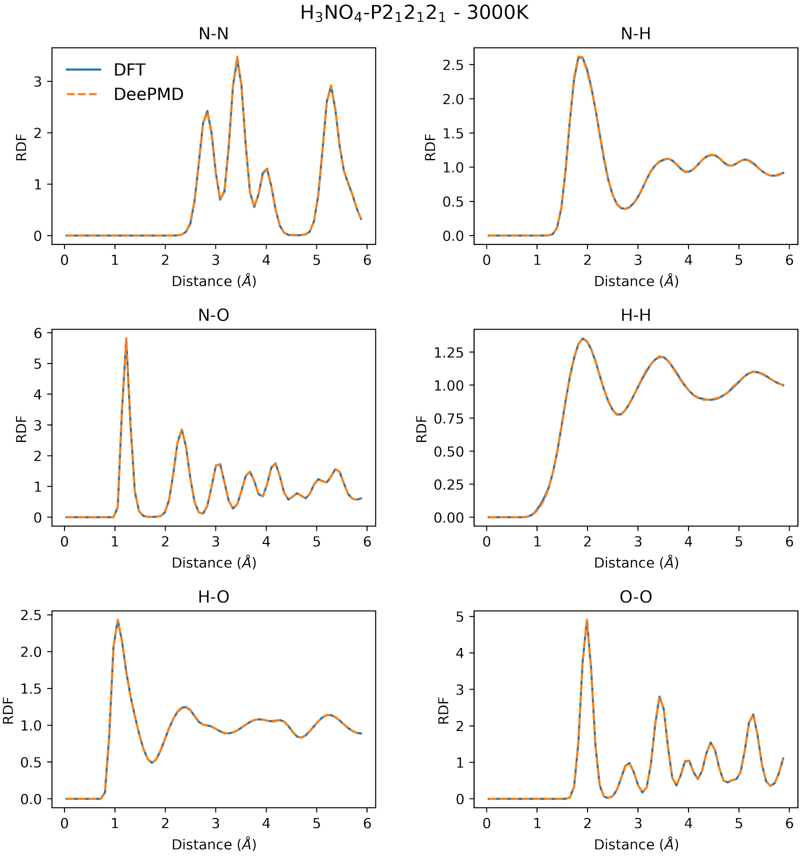

Figure S16 Radial distribution function comparison for DFT and DeePMD for H_3NO_4-$P2_12_12_1$ simulations for the hydrogen superionic phase, 3000 K at 6.033 g/cm^3.

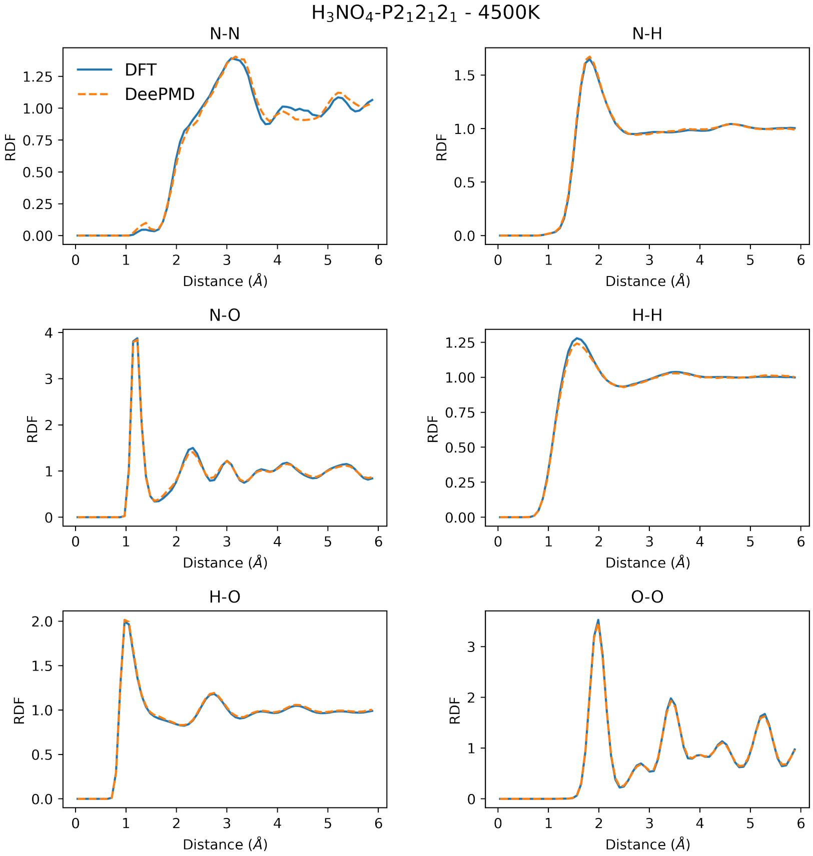

Figure S17 Radial distribution function comparison for DFT and DeePMD for H_3NO_4-$P2_12_12_1$ simulations for the doubly superionic phase, 4500 K at 6.033 g/cm^3.

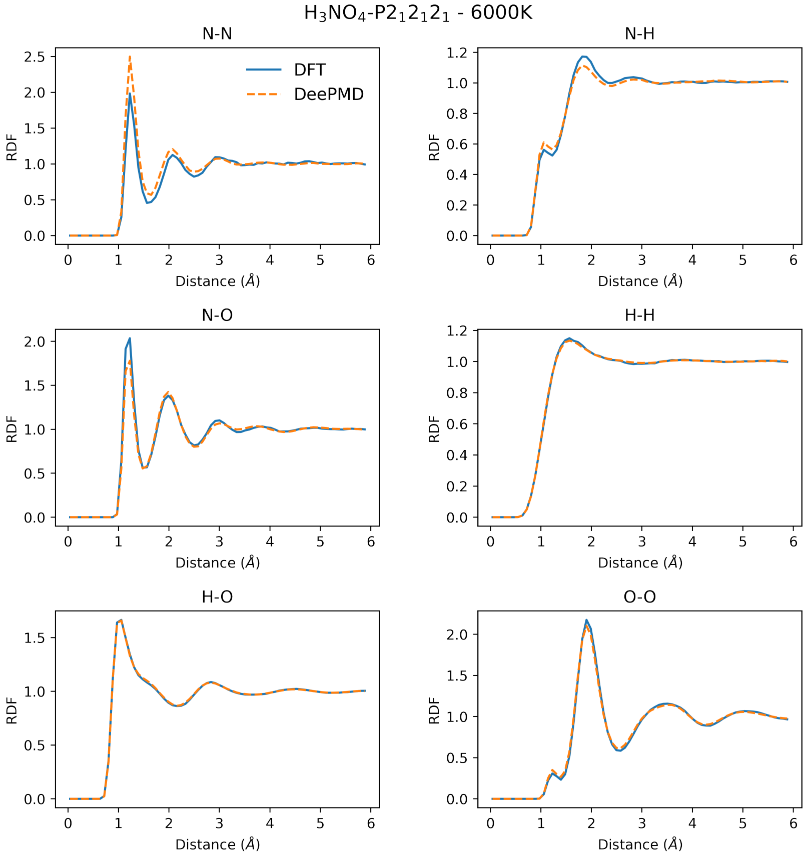

Figure S18 Radial distribution function comparison for DFT and DeePMD for H_3NO_4-$P2_12_12_1$ simulations for the liquid phase, 6000 K at 6.033 g/cm^3.

References

[1] Wang, H., Zhang, L., Han, J., Weinan, E.: DeePMD-kit: A deep learning package for many-body potential energy representation and molecular dynamics. Computer Physics Communications **228**, 178–184 (2018)

[2] Zhang, L., Han, J., Wang, H., Car, R., Weinan, E.: Deep potential molecular dynamics: a scalable model with the accuracy of quantum mechanics. Physical Review Letters **120**(14), 143001 (2018)

[3] Plimpton, S.: Fast Parallel Algorithms for Short-Range Molecular Dynamics. Journal of Computational Physics **117**(1), 1–19 (1995)

[4] Zhang, Y., Wang, H., Chen, W., Zeng, J., Zhang, L., Wang, H., Weinan,

E.: DP-GEN: A concurrent learning platform for the generation of reliable deep learning based potential energy models. Computer Physics Communications **253**, 107206 (2020)

[5] Cheng, B., Bethkenhagen, M., Pickard, C.J., Hamel, S.: Phase behaviours of superionic water at planetary conditions. Nature Physics **17**(11), 1228–1232 (2021)
