# Supplementary material for: Double superionicity in icy compounds at planetary interior conditions
Source: Nat Commun. 2023 Nov 21;14:7580. doi: 10.1038/s41467-023-42958-0 (PMC10663582; doi:10.1038/s41467-023-42958-0)
Supplement: Supplementary file 3 — Description of Additional Supplementary Files [file 41467_2023_42958_MOESM3_ESM.docx]

**Description of Additional Supplementary Files**

**File Name:** Supplementary Movie 1

**Description:** Superionic nitrogen diffusion through an FCC O lattice in H_3_NO_4_-P2_1_2_1_2_1_ at 4000 K for 5 ps. This cell had a total of 1920 atoms; H ions have been removed for clarity. Blue and red spheres represent nitrogen and oxygen ions, respectively, and the blue lines trace trajectories of N ions through the O sublattice.

**File Name:** Supplementary Movie 2

**Description:** Oxygen lattice crystallization during two-phase simulation of H_3_NO_4_-P2_1_2_1_2_1_ at 4000 K. N and H atoms are removed for clarity. Coloration of O ions indicates atomic symmetry, with green=FCC, red=HCP, blue=BCC, and white=other. At t=0, the middle of the cell is liquid and the outer edges are doubly superionic with an FCC O lattice. Within 5 ps, all O atoms in the liquid crystallize into an FCC lattice while the N atoms continue to diffuse throughout the cell.
